# Cells of renin lineage express hypoxia inducible factor 2α following experimental ureteral obstruction

**DOI:** 10.1186/s12882-015-0216-0

**Published:** 2016-01-08

**Authors:** Ania Stefanska, Diana Eng, Natalya Kaverina, Jeffrey W. Pippin, Kenneth W. Gross, Jeremy S. Duffield, Stuart J. Shankland

**Affiliations:** Department of Medicine, Division of Nephrology, University of Washington, Seattle, WA 98104 USA; Department of Molecular and Cellular Biology, Roswell Park Cancer Institute, Elm and Carlton Streets, Buffalo, NY 14263 USA; Biogen Idec, Cambridge, MA 02142 USA

**Keywords:** HIF-2α, UUO, Microvascular rarefaction, Pericytes, progenitor, Tubulointerstitial, PDGFßR, Fibrosis, Hypoxia

## Abstract

**Background:**

Recent studies indicate that mural cells of the preglomerular vessels, known as cells of renin lineage (CoRL), contribute to repair and regeneration of injured kidney glomeruli. However, their potential roles in tubulointerstitial disease are less understood. The aim of this study was to better understand CoRL number and distribution following UUO so that future mechanistic studies could be undertaken.

**Methods:**

We mapped the fate of CoRL in adult Ren1cCreER x Rs-tdTomato-R reporter mice that underwent UUO. Kidney biopsies from sham and UUO-subjected mice on days 3, 7, and 14 were evaluated by immunohistochemistry.

**Results:**

In sham animals, CoRL were restricted to juxtaglomerular location. At day 7 following UUO, CoRL increased two-fold, were perivascular in location, and co-expressed pericyte markers (PDGFßR, NG2), but did not express renin. At day 14 post UUO, labeled CoRL detached from vessels and were present in the interstitium, in areas of fibrosis, where they now expressed the myofibroblast marker alpha-smooth muscle actin. The increase in CoRL was likely due to proliferation as marked by BrdU labeling, and migration from the cortex. Following UUO starting from day 3, active hypoxia inducible factor-2α was detected in nuclei in labeled CoRL, in the cortex, but not those cells found in medulla.

**Conclusions:**

We have demonstrated that arteriolar CoRL are potential kidney progenitors that may contribute to the initial vascular regeneration. However, in chronic kidney injury (≥14 days post UUO), perivascular CoRL transition to myofibroblast-like cells.

**Electronic supplementary material:**

The online version of this article (doi:10.1186/s12882-015-0216-0) contains supplementary material, which is available to authorized users.

## Background

Unilateral ureteral obstruction (UUO), a commonly used experimental model of chronic kidney injury is characterized by tubular atrophy, inflammation and interstitial fibrosis [1, 2]. In UUO, the initiating damage is increased ureteral pressure transmitted retrograde to the kidney that causes secondary renal vasoconstriction and resultant reduced glomerular blood flow [3]. If the pressure is not relived, the renal vascular resistance remains increased, causing ischemia (reviewed in [4]). The resultant tubulointerstitial fibrosis in UUO is multi-factorial, including interstitial macrophages producing pro-inflammatory cytokines, tubular cells undergoing apoptosis, and resident renal cells transitioning to collagen-producing cells [1]. The origin of the collagen producing cells has been attributed to the perivascular cell population, namely pericytes and perivascular fibroblasts. Following UUO, perivascular cells become activated, and detach from the underlying vessels. The consequences of pericyte detachment include that endothelial cells are deprived of survival factors [5], vascular tubes become unstable and more permeable leading to microvascular rarefaction [6], and migrating perivascular cells can de-differentiate into myofibroblasts, thereby becoming a source of collagen [7, 8].

Less is understood the reparative, or attempted reparative processes, if any, in UUO. Cells of renin lineage (CoRL) refer to all possible cellular derivatives originating from renin expressing cells at time captured by reporting. Fate tracking studies showed that in development, CoRL give rise to juxtaglomerular (JG) cells producing renin and to non-renin-producing cells such as smooth muscle cells, mesangial cells, tubular cells and extrarenal cells [9]. Recently, the lineage relationship between Ren^+^ and Foxd1^+^stromal cells was clarified when it was revealed that all mural cells, including renin cells, are derived from Foxd1 stromal cells [10]. Studying CoRL (Ren^+^ progenitors) provides an opportunity to characterize in depth a subpopulation of stromal cells. It is important because we need better understanding of different subpopulation of stromal cells to design specific therapeutic interventions [11].

Pericytes have gained recognition in the kidney for their role in pathogenesis of fibrosis [7, 8]. Interestingly, CoRL have been acclaimed as a candidate progenitor of the kidney. Several studies showed that CoRL can regenerate mesangial cells [12, 13], podocytes [14], parietal epithelial cells [14], and erythropoietin-producing cells [15]. The most recent study from our lab demonstrates that in glomerular injury and remnant kidney models, CoRL migrate to the interstitium and regenerate into pericytes [16]. The current study was designed to further explore the role of CoRL in progressive tubulointerstitial injury, the experimental model of unilateral ureteral obstruction.

## Methods

### Animals

To study the fate of renin lineage cells in chronic kidney disease, we used RenCreER (Ren1cCreERxRs-tdTomato) transgenic mice on a mixed C57 BL10/C3H background [16]. In RenCreER mice, cells of renin lineage are only labeled permanently in inducible manner with tdTomato red protein within temporal windows defined by the administration of tamoxifen. 8–9 week-old mice were given Tamoxifen (100 mg/kg) by IP injection for 6 days on alternate days, as we have previously reported [16, 17]. We waited at least 7 weeks between giving tamoxifen and inducing the disease model, to allow significant washout of tamoxifen to exclude possibility of recombination in other cell types. Animal protocols were approved by the University of Washington Institutional Animal Care and Use Committee (2968-04).

### Experimental model of kidney fibrosis

Unilateral ureteral obstruction (UUO) was performed in adult female mice, as previously described [7, 18, 19]. Briefly, mice were anesthetized with isofluorane (1 %, inhaled). UUO was induced by left ureteral ligation using a 4-0 silk tie suture at two points. Sham operated mice underwent the same procedure except that left ureter was only exposed by flank incision, served as controls (*n* = 6). Kidneys were harvested on d3 (*n* = 6), d7 (*n* = 17), and d14 (*n* = 6).

### Assessment of kidney fibrosis

Histological analysis of fibrosis was performed on fixed renal tissue, embedded in paraffin, and sectioned at a thickness of 4 μm. Connective tissue deposition was examined with Picrosirius Red Stain Kit (Polysciences, Inc, Warrington, PA, USA) and collagen I (1:100, Millipore, Billerica, MA, USA), staining, as we have previously described [20–22]. Additionally, co-staining of CoRL and a myofibroblast marker alpha smooth muscle actin (αSMA, 1:10.000; Sigma, Saint Louis, MI, USA) were performed to determine whether CoRL become myofibroblasts in kidney fibrosis.

### Detection of tdTomato reporter

In order to visualize tdTomato reporter labeling used to label CoRL in RenCreER mice, kidneys were fixed in 10 % buffered formalin, embedded in paraffin, and sectioned at a thickness of 4 μm. Kidney sections underwent deparaffinization, heat-mediated antigen retrieval in citrate buffer pH 6.0, and blocking unspecific background (Accurate, San Jose, CA, USA). Immunofluorescent staining for the tdTomato reporter was performed with DyeLight 594-conjugated RFP (Red Fluorescent Protein) rabbit antibody (1:100, Rockland Immunochemicals for Research, Gilbertsville, PA, USA) at room temperature for 1.5 h. tdTomato fluorescent signal is discernable in non-paraffin- embedded tissue only.

### Identification of cell proliferation

BrdU (5-Bromo-2-deoxyridine) incorporation assay was used to quantitate cell cycle entry and proliferation. 10 μl of BrdU (Amersham Cell Proliferation Labeling Reagent, GE Healthcare Life Sciences, Little Chalfont, UK) per gram body weight was administered via IP on alternate days following UUO. Double immunostaining was performed for BrdU and tdTomato. Antibody paraffin embedded tissue was prepared and blocked as described above. Avidin/biotin blocking (Vector Laboratories, Burlingame, CA, USA) was performed to block endogenous biotin and prevent unspecific staining while using biotin-streptavidin labeling system. Tissue was incubated overnight at 4 °C with a primary mouse anti-BrdU (1:200, Amersham, GE Life Sciences, Buckinghamshire, UK). This was followed with a biotinylated goat anti-mouse antibody (1:500, Jackson Immunoresearch Laboratories, Inc, West Grove, PA, USA) incubated at room temperature for 1 h. The signal was amplified by incubation with streptavidin-conjugated with Alexa Fluor 488 (1:100; Invitrogen, Grand Island, NY, USA) for 45 min. Negative control staining was performed by omitting primary antibody staining.

### Assessment of vascular changes

Endothelial marker staining CD31 (PECAM-1) was performed to examine changes in microvascular density, and to demonstrate any perivascular location of CoRL and pericytes. Rat anti-mouse CD31 (1:100; Dianova, Hamburg, Germany) was incubated overnight at 4 °C following incubation with secondary anti-rat antibody conjugated with Alexa Fluor 647 (1:100 Invitrogen).

Pericytes were identified by the expression of NG2 and PDGFRß. To identify pericytes derived from CoRL, triple imunostaining was performed on frozen tissue sections as follows. Rabbit anti-NG2 antibody (1:100; Millipore, Billerica, MA, USA) was incubated overnight at 4 °C, followed by biotinylated anti-rabbit antibody (1:500; Vector) incubation at room temperature for 1 h, and streptavidin conjugated with Alexa Fluor 647 (1:100; Invitrogen, Grand Island, NY, USA) for 45 min. To prevent non-specific staining for the primary antibodies from the same species pre-incubation with anti-rabbit IgG Fab (1:25; Jackson ImmunoResearch Laboratories, West Grove, PA, USA) was followed by rabbit IgG Fab incubation (1:25; Jackson ImmunoResearch Laboratories). Rabbit anti-PDGFRß antibody (1:100; Abcam, Cambridge, MA, USA) was incubated with tissue sections overnight at 4 °C. Secondary donkey anti-rabbit antibody conjugated with Alexa Fluor 488 (1:100, Invitrogen) was incubated for 1 h at room temperature. Finally, anti-tdTomato antibody was applied.

To examine hypoxia-activated locations in UUO, HIF-2α (hypoxia inducible factor-2 α) staining was performed together with tdTomato reporter staining on frozen tissue sections. Rabbit anti-HIF-2α antibody (1:200; Novus Biological, Littleton, CO, USA) was incubated overnight at 4 °C, followed by biotinylated anti-rabbit antibody (1:500; Vector) incubation at room temperature for 1 h, and streptavidin conjugated with Alexa Fluor 647 (1:100; Invitrogen) for 45 min. Positive staining was assessed based on nuclear HIF-2α localization confirmed by DAPI staining.

### Image analysis and statistical analysis

Reporter positive-, BrdU- stainings were quantified on 20 images of kidney cortex/medulla using 200x total magnification. Fluorescent imaging was performed using EVOS®FL Cell Imaging System (Life Technologies). One-way ANOVA with Bonferroni post hoc test was used to compare groups with P ≤ 0.05 as a criterion for statistical significance. Data were presented as means ± SEM. All data were analyzed in GraphPad Prism 5.0 (GraphPad Software, La Jolla, CA, USA).

## Results

### UUO results in microvascular rarefaction and increased collagen staining

Additional file 1: Figure S1A shows the expected classical hydronephrosis in the obstructed kidney as reported by others [1, 4], with compensatory growth in the non-obstructed kidney. Prolonged ureteral obstruction leads to renal parenchymal damage and as a consequence loss of kidney mass. Following UUO, there was a decrease of the ligated/non-ligated kidney weight ratio d7 (1.00 ± 0.04 vs. 0.79 ± 0.3, *p* < 0.01 vs. sham kidney) and d14 (1.00 ± 0.04 vs. 0.64 ± 0.07, *p* < 0.001 vs. sham kidney) (Additional file 1: Figure S1B).

Loss of microvessels affects oxygen supply and toxin removal [23]. In chronic kidney disease, microvascular rarefaction contributes to tubulo-interstitial scarring and is a potential therapeutic target [19, 24]. Following UUO, there was a progressive decline in endothelial CD31 staining in the cortex and medulla on d7 and d14 compared to the sham kidney (Additional file 1: Figure S1C). Picrosirius Red staining labels collagen I and III fibers. As expected, interstitial collagen levels were very low in sham kidneys (Additional file 1: Figure S1D). Following obstruction, there was a progressive increase of picrosirius red staining on days 3, 7, and 14 (Additional file 1: Figure S1D). The percentage area of Picrosirius Red staining increased from 3.05 ± 1.02 % in sham kidney to 5.61 ± 0.64 on day 3 (*P* > 0.05), through 9.86 ± 1.48 on day 7 and was the highest on day 14 (14.03 ± 2.8, *p* < 0.01 vs. sham, and *p* < 0.05 vs. day 3) (Additional file 1: Figure S1E). Taken together in this mouse strain, the decrease in endothelial cell staining and increase in fibrosis are consistent with classic interstitial changes in UUO.

### Interstitial CoRL number increases in UUO

#### Cortical changes

**Fig. 1:**
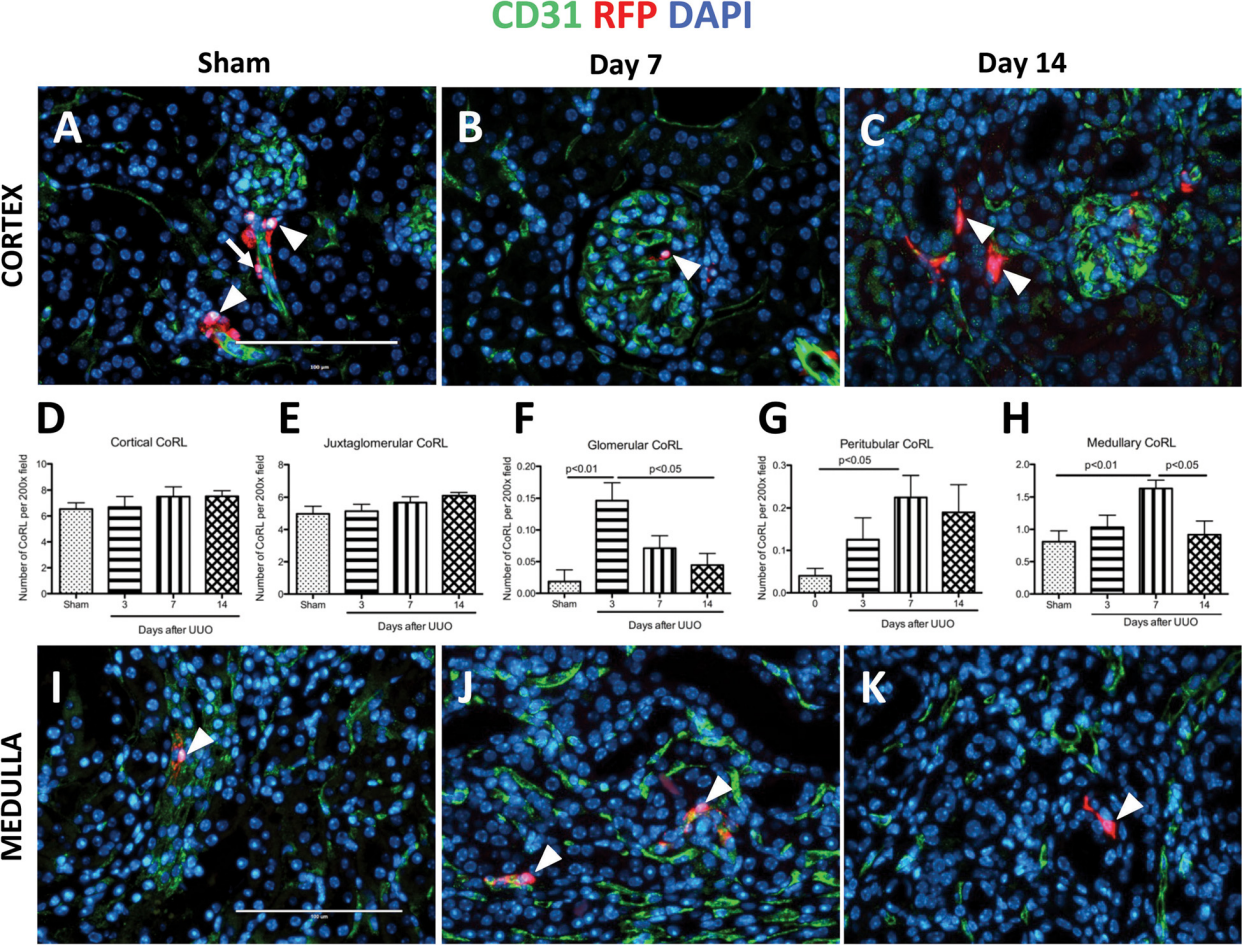
The number of cells of renin lineage (CoRL) increases beyond the JG location following UUO. CoRL were identified by RFP reporter staining (red), endothelial cells were labeled with CD31 staining (green). Nuclei are labeled with DAPI (*blue*). (**a**–**g**) CoRL in Cortex. (**a**) Representative images show that in sham-operated mice, labeled CoRL (*arrowheads*) are predominantly localized to the juxta-glomerulus, with fewer detected along afferent arterioles (*arrow*). (**b**) At d7 post UUO, occasional labeled CoRL were detected in glomerular capillary loops. (**c**) Labeled CoRL were rarely detected in peritubular locations at day 14. Quantification: (**d**) Total cortical CoRL number was not statistically significant following UUO, (**e**) the same was found when CoRL were quantified exclusively in JG compartment. However, (**f**) there was a significant increase of intraglomerular CoRL at d3, and (**g**) peritubular CoRL at d7 and d14. (**h**–**k**) *CoRL* in Medulla. (**h**) Quantification - total CoRL number in the medulla was significantly increased on day 7 following UUO. (**i**) In the medulla of the sham kidney, CoRL were restricted to vasa recta. (**j**) Following UUO there was an increase of CoRL in vasa recta on day 7 (*arrowheads*). (**k**) 14 days after UUO, a subset of CoRL localized away from any vessels, being present in the interstitium

**Fig. 2:**
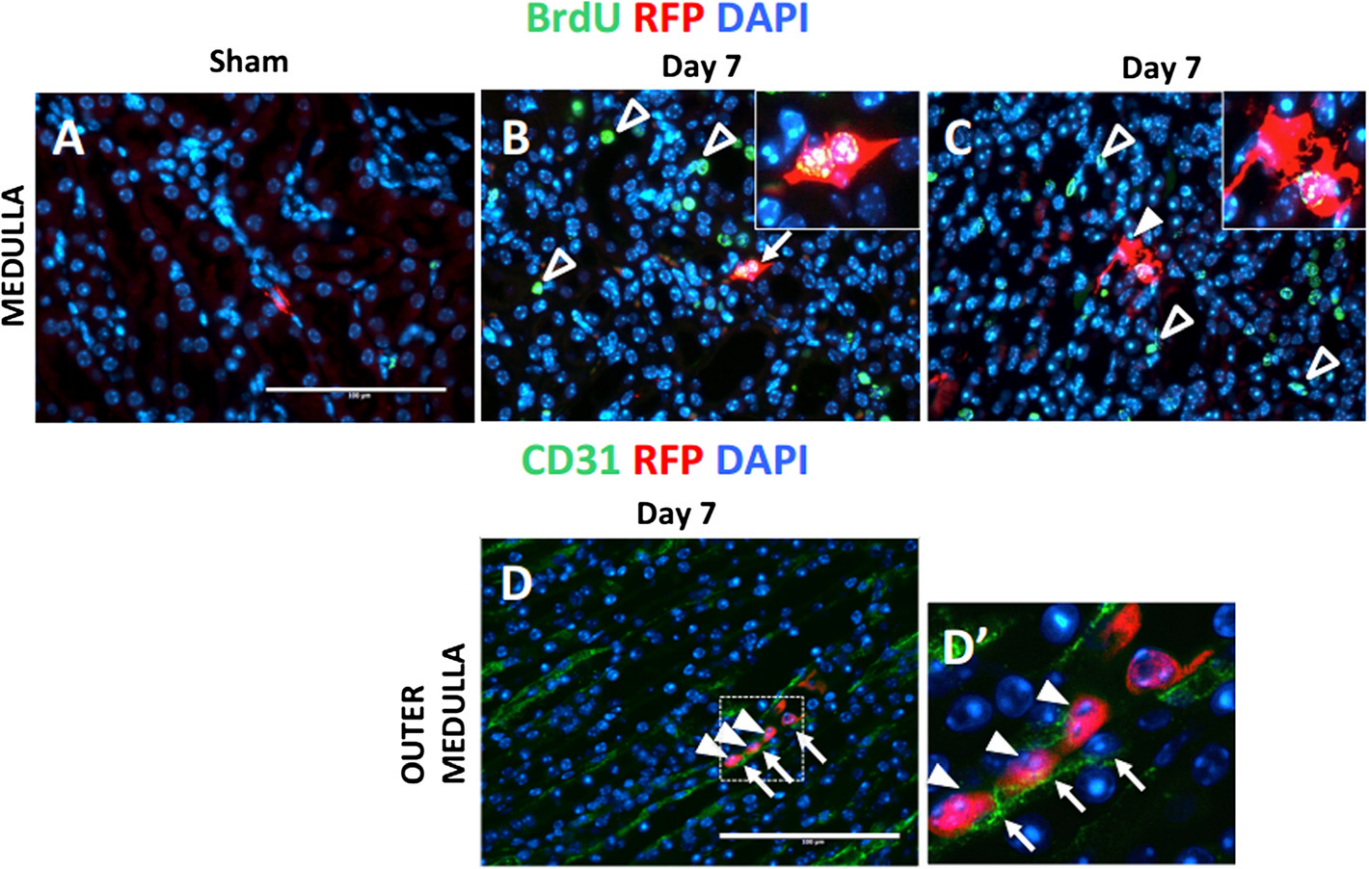
Proliferative and migratory phenotype of CoRL in UUO. (**a**–**c**) To detect proliferation in CoRL, double-staining was performed for Red fluorescent protein (*red*) to identify CoRL, and BrdU (*green*) to examine cell proliferation. Nuclei are labeled with DAPI (*blue*). Medulla: (**a**) CoRL did not co-express BrdU in sham kidneys. ( **b**) At day 7 post UUO, a subset of CoRL co-expressed BrdU (*yellow color*; arrow shows example). The inset demonstrates this at higher magnification. As expected, other peritubular cells stained for BrdU (*open arrowheads*). (**c**) Some dual CoRL^+^/BrdU^+^ cells displayed elongated shape with long cellular processes suggesting cell motility when viewed at high power (inset). (**d**) To better define the subset of labeled CoRL that appeared to be migrating based on their shape (identified by RFP staining, arrowheads), double staining was performed for the endothelial cell marker CD31 (*green, arrows*). (**d’**) On occasion, CoRL were detected within vessels in the medulla

Double-staining was performed for RFP (to mark labeled CoRL), and CD31 (endothelial cell marker). Similar to what we have previously reported [16], when CoRL reporter mice are given tamoxifen to induce permanent CoRL reporting, and then undergo a sham procedure as control, labeled CoRL (RFP positive, red color) in the cortex are typically localized and restricted to juxtaglomerular (JG) compartment (Fig. 2a, arrowheads) and along afferent arterioles (Fig. 1a, arrow). Following UUO, CoRL were also detected within the capillary loops of an occasional glomerulus (Fig. 1b), and also in peritubular locations (Fig. 1c).

In UUO there were no significant changes in the number of total cortical CoRL (including JG, afferent arterioles, peritubular and glomerular CoRL) (Fig. 1d). There was a slight trend in the CoRL number increasing in the JG after two weeks of kidney injury, however these changes were not significant (Fig. 1e). Interestingly, at d3 post UUO, glomerular CoRL number increased compared to sham kidney (0.02 ± 0.02 vs. 0.15 ± 0.03, *p* < 0.01 vs. d3), and d14 (0.04 ± 0.02 vs. d3, *p* < 0.05) (Fig. 1f). In contrast, peritubular CoRL number increased significantly at d7 (0.23 ± 0.05 vs. 0.04 ± 0.02, *p* < 0.05 vs. sham), (Fig. 1g). On d3 (0.13 ± 0.05, *p* > 0.05) and d14 (0.19 ± 0.07, *p* > 0.05) following UUO, there were trends towards an increase in peritubular CoRL number (Fig. 1g).

#### Medullary changes

In the medulla of sham kidneys, labeled CoRL were detected in vasa recta (Fig. 1i). At d7, increased RFP labeled CoRL were typically adherent to underlying vessels (Fig. 1j). Noteworthy was that at d14, the majority of labeled CoRL were no longer adherent to underlying vessels, but rather were detected outside the vascular bed (Fig. 1k). Following UUO, there was a trend to an increase in the number of medullary CoRL at d3 (1.03 ± 0.19 vs. 0.81 ± 0.17, *p* > 0.05 vs. sham). However, there was a significant increase in medullary CoRL on d7 (1.62 ± 0.13, *p* < 0.01 vs. sham). The number of labeled CoRL was lower at d14 post UUO (0.92 ± 0.21, *p* < 0.05 vs. d7), and was no different from sham kidneys (*P* > 0.05) (Fig. 1h).

These results show that the number of labeled CoRL increases in peritubular locations in both cortex and medulla on d7. By d14, the majority of labeled (almost 100 %) CoRL had detached from their vasculature, raising the possibility of an ultimate myofibroblast fate.

### CoRL proliferate and migrate following UUO

Having observed increased CoRL number in the medulla following UUO, we next asked whether this was due to cell proliferation by administering repeated BrdU pulses. As expected, there was an increase of tubular cell BrdU staining following UUO, used as an internal positive control (Fig. 2b, c, open arrowheads). In the medulla, CoRL BrdU staining was only detected on UUO day 7 (Fig. 2b). Of the total medullary CoRL number, 3.78 ± 5.28 % stained for BrdU. Several proliferating medullary CoRL exhibited a marked shape change that gave the appearance of a migratory phenotype (Fig. 2c, arrowhead). Moreover, using CD31 staining to highlight endothelial cells, occasional labeled cells were detected within the lumen of medullary vessels (Figs. 2d, d’). These data show that CoRL proliferation was not detected in the cortex, including their original location in the juxta-glomerulus. However, a subset of CoRL that had migrated did proliferate when in the medulla.

### Renin is not expressed in interstitial CoRL

At 70 days postnatal in a mouse, renin expression is restricted to the JG [25]. Because our data showed an increase of interstitial CoRL (i.e. beyond the JG compartment) we next determined if these cells retained renin protein. Double staining for renin and the RFP reporter shows that in both sham (not shown) and obstructed kidneys, renin staining was restricted to cells in the JG only (Additional file 2: Figure S2A and B). These results support the concept that renin lineage cells, when challenged, can take on different cell phenotype and in doing so, no longer express renin [15, 26].

### CoRL co-express pericyte markers following UUO

Both CoRL and pericytes derive from Foxd1 lineage cells [10]. CoRL are primarily located in the JG compartment and afferent arterioles, whereas renal pericytes are typically located around peritubular capillaries and vasa recta [27, 28]. Foxd1 lineage tracking demonstrated that pericytes and perivascular fibroblasts are major cellular contributors to kidney fibrosis [7, 8]. To determine if CoRL contribute to pericyte recruitment following a compromised microvasculature post UUO, triple staining was performed for the reporter (RFP), pericyte markers (PDGFßR, NG2).

**Fig. 3:**
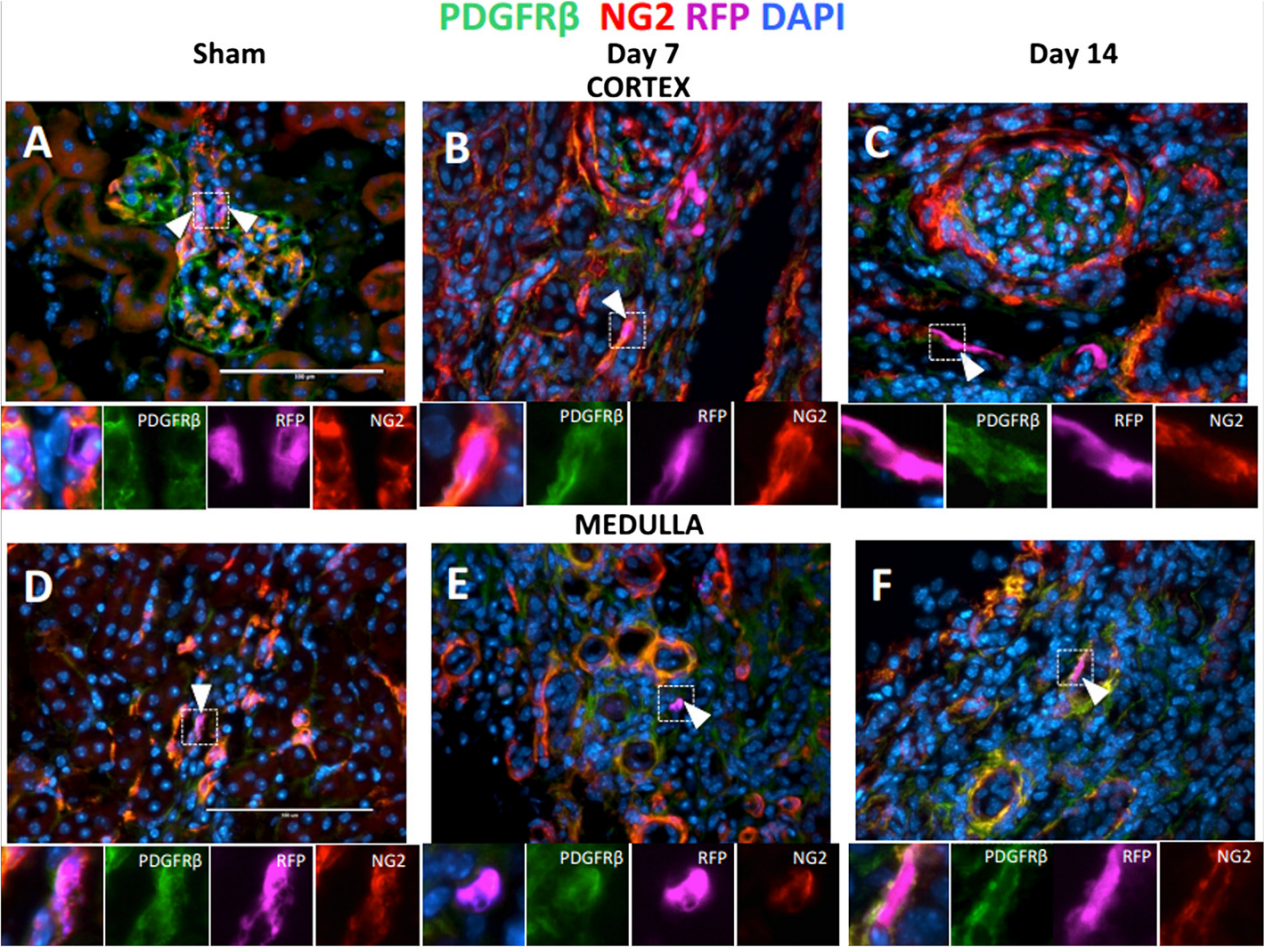
Interstitial CoRL co-express pericytes markers. To label pericytes, antibody staining against PDGFRß (*green*) and NG2 (*red*) was performed, CoRL were identified by RFP staining, nuclei are labeled with DAPI. (**a** *–* **c**) Cortex: (**a**) Representative images show that in sham-operated mice, CoRL co-express pericyte markers in JG (*arrowhead*). Insets show high power images of juxtaglomerular CoRL. In UUO, there was an accumulation of interstitial pericytes (*orange color*). Interstitial CoRL were found in peritubular areas co-expressing pericyte markers at (**b**) 7 day post UUO, and (**c**) 14 days post UUO. Insets show high power images of CoRL. (**d**–**f**) Medulla: (**d**) Sham medulla shows CoRL co-expressing pericyte markers in vasa recta (arrowheads). Insets show high power images. Pericyte marker staining increases in UUO (*orange color*). Interstitial CoRL were co-labeled with pericyte markers at (**e**) day 7 post UUO, (**f**) day 14 post UUO. Insets show high power images of interstitial CoRL

Staining for endogenous pericyte markers was overall increased in UUO, similar to published data regarding pericytes accumulation in fibrosis [7, 8, 29]. In the sham kidney, NG2^+^/PDGFRß^+^ staining was detected in mesangial cells, afferent arterioles, peritubular capillaries, and vasa recta (Fig. 3a, d). At d3 post UUO there was an increase of interstitial PDGFRß expression, whereas NG2 staining was fainter in both cortex and medulla (not shown). However, at d7 post UUO, there was an increase of staining for both PDGFßR and NG2 (Fig. 3b, e), which persisted at d14 (Fig. 3c, f). Triple staining revealed that labeled CoRL (identified by RFP staining) in the interstitium co-expressed both PDGFßR and NG2 at days 3, 7 and 14 post UUO. The aforementioned results show that labeled CoRL always co-express pericyte markers and this is consistent with them likely being truly pericytes. In such a capacity, CoRL may indeed participate in vessel remodeling in UUO.

### A subset of pericytes that have detached from vessels express increased αSMA

To determine if CoRL become myofibroblasts and localize to scarred areas, triple staining was performed for RFP (CoRL reporter), αSMA (a marker of VSMC which appears *de novo* in myofibroblasts), and CD31 (endothelial marker).

#### Cortical changes

**Fig. 4:**
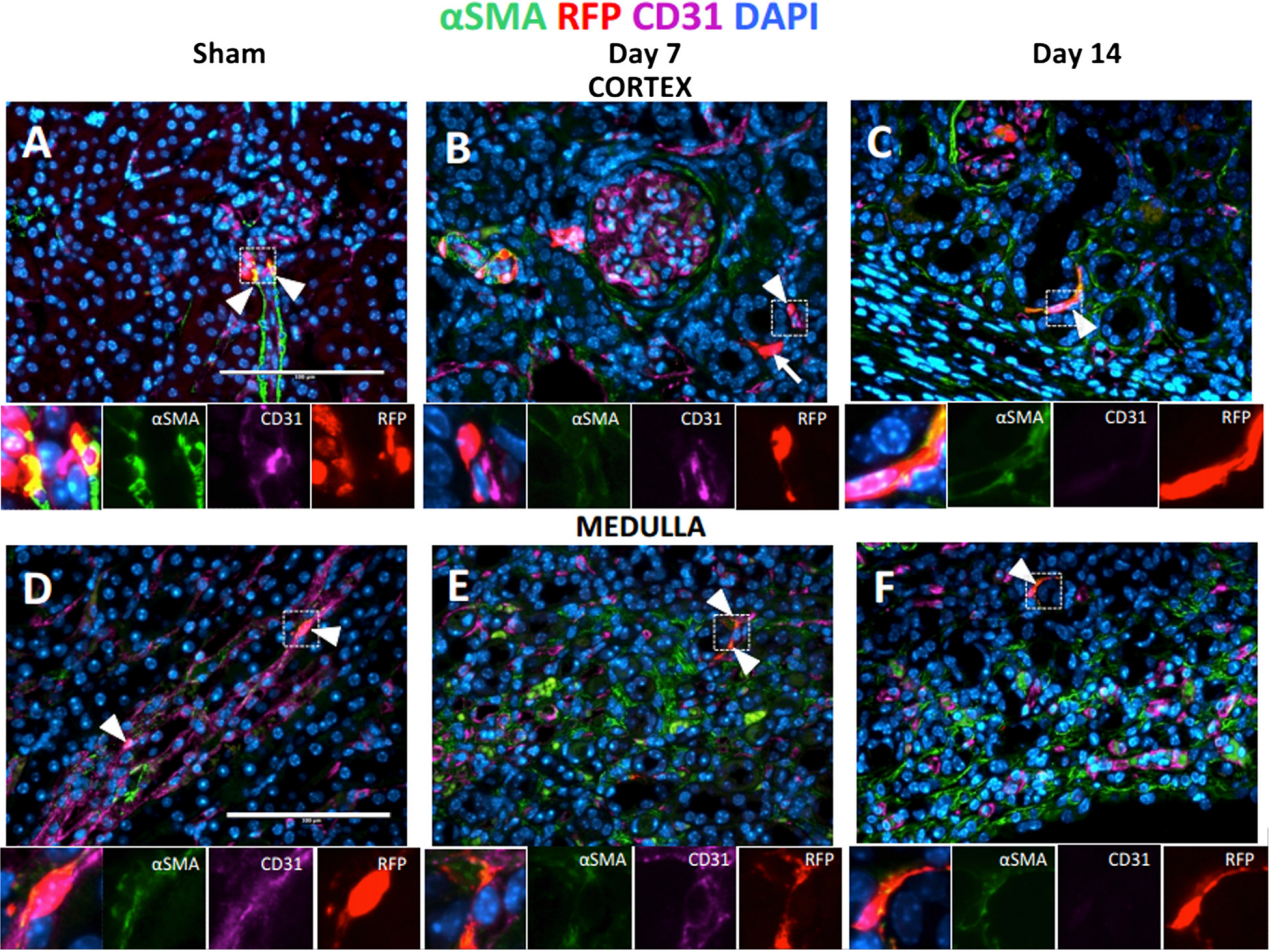
αSMA is expressed by interstitial CoRL in fibrosis. Red fluorescent protein (*red*) staining identified CoRL, αSMA (*green*) staining was used to mark vascular smooth muscle cells and myofibroblasts, CD31 staining denotes endothelial cells (*magenta*). Nuclei are labeled with DAPI (*blue*). (**a**) In the sham cortex, CoRL were found in JG compartment (arrowheads, insets). (**b**) At d7 post UUO, CoRL were localized to perivascular area (arrowhead, insets), however, some CoRL were no longer supporting microvessels (*arrow*). (**c**) At 14 days post UUO, majority of CoRL (*arrowhead*) were found outside vascular bed and expressing αSMA suggesting the myofibroblastic conversion. Insets show high power images of merged and single stained panels (**d**) In the sham medulla, CoRL were found in vasa recta (arrowhead) and co-expressing αSMA. (**e**) 7 days following UUO, CoRL were still found surrounding endothelial cells (*arrowhead, insets*). (**f**) 14 days post UUO, CoRL and localized to avascular areas and expressing αSMA (*arrowhead*). Insets show high power images of merged and single stained panels

In the sham kidney cortex staining for αSMA and CoRL is present in afferent arterioles (Fig. 4a, insets, arrowhead). Post UUO, all interstitial CoRL in the cortex co-expressed αSMA at days 3, 7, 14. At d3, CoRL displayed perivascular location (not shown). In addition to peri-endothelial location (Fig. 4b, insets, arrowhead), CoRL were also found outside capillary bed at d7 post UUO (Fig. 4b, arrow). 2w post UUO when CoRL had migrated away from the vessels, they continued to express αSMA, suggesting a myofibroblast nature (Fig. 4c, insets, arrowhead).

#### Medullary changes

In the sham kidney medulla, CoRL are present in vasa recta only, and these cells co-express αSMA (Fig. 4d, insets, arrowhead). Following UUO, interstitial CoRL co-express αSMA on days 3, 7 and 14. At d7 CoRL were detected occasionally in extravascular locations (not shown), while also being peri-vascular (Fig. 4e, insets, arrowheads). By co-staining with CD31 we confirmed that the majority of CoRL (almost 100 %) that had detached from the underlying vessels on d14 are αSMA-expressing myofibroblasts (Fig. 4f, insets, arrowheads).

### HIF-2α is activated in CoRL in the JG compartment and in afferent arterioles in UUO

Hypoxia secondary to vasoconstriction and microvascular rarefaction is an important mechanism mediating tubulointerstitial injury [30]. In the kidney, HIF-2α is activated by hypoxia in interstitial fibroblasts-producing erythropoietin [31] and in endothelial cells [32, 33]. HIF stabilization and permanent hypoxic state can be achieved in a mouse model by Vhl deletion. Targeted deletion of Vhl in the cells of renin lineage demonstrates HIF-2α expression along the afferent arterioles, glomerular vascular poles, and intraglomerular cells [15]. Therefore, cells in the juxta-glomerulus and afferent arterioles respond to hypoxia by activating HIF-2α. To examine changes in HIF-2α might in CoRLs following UUO, co-staining was performed for RFP (CoRL label) and HIF-2α.

#### Cortical changes

**Fig. 5:**
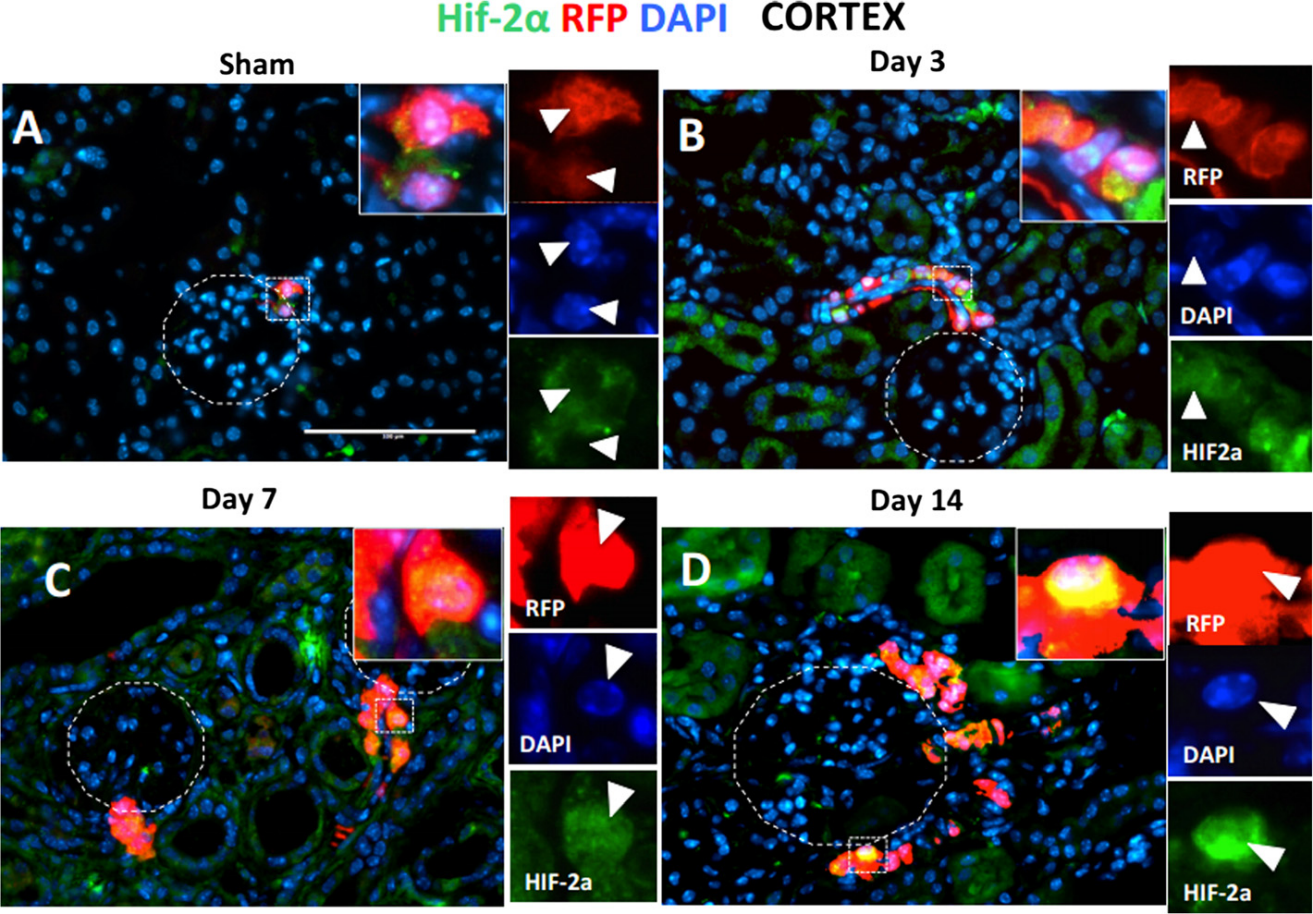
Nuclear translocation of HIF-2α in JG and afferent arteriole in UUO. Red fluorescent protein (*red*) staining identified CoRL, HIF-2α (*green*) staining was used to detect hypoxia-activated cells. Nuclei are labeled with DAPI (*blue*). In the sham kidney cortex, there was (**a**) a cytoplasmic HIF-2α staining in JG cells (inset shows single panel colors, arrowheads indicates the same cell). In UUO kidney, there was an accumulation of HIF-2α staining in the nuclei in afferent arterioles and JG (insets show single panel colors, arrowheads indicates the same cell) on (**b**) day 3, (**c**) day 7, (**d**) day 14 following kidney injury

In sham-operated kidneys, HIF-2α staining was detected in the cytoplasm of a subset of labeled CoRL in the JG compartment (Fig. 5a, inset, arrowheads). HIF-2α staining in the cytoplasm is considered the non-active form, whereas when active, HIF-2α translocates to the nucleus [34]. Following UUO on days 3, 7 and 14, HIF-2α staining was detected in the nucleus (co-localized with DAPI) in labeled CoRL in the JG compartment and afferent arterioles (Fig. 5b–d, insets, arrowheads). These subcellular changes in the location of HIF-2α staining were consistent with the presence of the inactive form in sham kidneys, but the active form in labeled CoRL post UUO in the JG compartment.

#### Medullary changes

In the sham kidney medulla, HIF-2a was not detected in labeled CoRL (Additional file 3: Figure S3A) In contrast to the cortex, HIF-2α never localized to labeled CoRL in the medulla post obstruction. Thus was not a false negative result because following UUO there was a progressive increase in HIF-2α staining of interstitial cells of non-CoRL type (Additional file 3: Figure S3B, C, and D, arrowheads).

## Discussion

UUO is characterized by hypoxia, interstitial vascular loss and progressive kidney fibrosis. The compensatory mechanisms that might attempt to counter these events are not well understood. We demonstrated that following UUO, the number of medullary cells of renin lineage (CoRL) number increases due to cell proliferation and migration. Although CoRL initially appear to be reparative of interstitial microvessels following UUO by transdifferentiating in to pericyte-like cells, they are likely ultimately injurious by transdifferentiating into myofibroblast-like cells. These changes are preceded by activation of HIF2a in CoRL in the juxta-glomerular compartment following UUO.

The first major finding from these studies was that the number of CoRL increases following UUO. We used inducible Ren1cCreER xRs-tdTomato-R reporter mice to fate map a subset of CoRL that were permanently labeled specifically during the period of tamoxifen induction. The distribution of cells-expressing renin is very different during development compared to adults [9]. Therefore, labeling mice at 7–8 weeks of age focuses on adult CoRL that derive from the juxtaglomerular (JG) compartment.

We next asked how might CoRL number increase following UUO? A first consideration was migration, from their original location in the JG to the medulla. Several lines of evidence help support this. First, using an inducible reporter system, labeled CoRL in the cortex were restricted to the JG. Thus, the presence of labeled CoRL in the intracapillary loops of some glomeruli, and in the cortical interstitium is in keeping with them migrating. Second, many CoRL in the cortex and medulla had an elongated shape, reminiscent of a migrating cell. Third, several interstitial vessels contained labeled CoRL within their lumens. Proliferation is another mechanism that might explain the twofold increase in medullary CoRL post UUO. To this end, we frequently performed BrdU pulse chases to maximally capture cell proliferation. BrdU staining was detected in a subset of CoRL in the cortex and medulla, but not in the JG. Moreover, several of CoRL with elongated shapes stained for BrdU. These data suggest that once CoRL had moved from the JG, a subset proliferated. However, we cannot exclude the possibility that native CoRL in the medulla did not proliferate, not migrate. Taken together, the increase in medullary CoRL post UUO is likely due to a combination of proliferation in CoRL that had migrated away from the JG, migration of non-proliferating CoRL and proliferation of pre-exisiting CoRLs in the medulla.

The second major finding in these studies was the transdifferentiation of medullary CoRL in to pericytes or myofibroblasts. Several lines of evidence show that CoRL have marked plasticity under certain conditions [13–16]. Accordingly, we next asked what, if any, cell type did CoRL transdifferentiate into in the medulla following UUO. The first clue that they likely were transdifferentiating was the loss of their endocrine function by virtue that they no longer expressed the renin protein. Second, because at d7 labeled CoRL were largely confined to surrounding interstitial vessels, we explored the possibility that a subset were transdifferentiating in to pericytes. Indeed, non-renin expressing labeled CoRL co-expressed the pericyte markers NG2 and PDGFRß. Indeed, CoRL have been reported to transdifferentiate into pericytes during development [9] and in glomerular disease [16]. We can only speculate that the transdifferentiation of a subset of CoRL to pericytes early in disease is an attempt to maintain or even replace native pericytes, and thus the interstitial vasculature.

Detached pericytes can differentiate into collagen-producing myofibroblasts [7, 19]. Here, we report that a subset of CoRL later underwent further changes to that more consistent with a myofibroblast. At day 14 post UUO, the majority of labeled interstitial CoRL were away from any blood vessels. Because these areas were typified by interstitial fibrosis, we asked if CoRL were transdifferentiating into myofibroblasts. Indeed, a subset of CoRL did begin to express aSMA, suggesting that they acquired a pro-fibrotic phenotype. It is not clear which of the following scenarios occurred first: a decrease in interstitial vessels (i.e. reduced CD31 staining) forced pericyte-like CoRL to detach, and/or if the detachment of pericyte-like CoRL lead to unhealthy underlying vessels, followed by rarefaction. Regardless, it is likely that similar to other native cells in the interstitium that acquire myofibroblast-like features, the subset of CoRL doing so also likely contribute to the increased fibrosis later in UUO. Finally, our data are in agreement with the current view that Foxd1 lineage cells contribute to fibrosis [35] especially that CoRL have been recently shown to derive from Foxd1^+^ cells [10].

Hypoxia is a common pathway for chronic kidney disease, including UUO [36, 37]. During kidney obstruction, renal blood flow is reduced as a consequence of pre-glomerular vessel constriction that in turn impairs post-glomerular/peritubular perfusion [38]. HIF-2α is activated in interstitial and endothelial cells in hypoxia [32, 33]. Extracellular matrix deposition further propagates hypoxia to the tubulointerstitium since it increases the distance between capillaries and tubules, decreasing oxygen diffusion [39]. In general, HIF-1 and -2 are both activated in hypoxia, but in a different manner: HIF-2 is activated in mild hypoxia <5 % O_2_ for many hours (still upregulated after 72 h), whereas, HIF-1 is rapidly induced at 1 % O_2_ to mediate acute responses, and declines to the low levels within 72 h [40]. This suggests that HIF-2α has a role in the adaptation to chronic hypoxia, where it is the main regulator of erythropoietin production [41], vascular tumorigenesis [42], cell proliferation [43], and vessel remodeling in disease [44, 45]. Hypoxia is also associated with stem cell phenotype, pluripotent stem cell culture at hypoxic conditions (5 % O_2_) stabilize HIF-2α, increase proliferation and stem cell marker expression [46].

The third major finding was that HIF-2α, a hypoxia-activated factor, is induced in CoRL in the JG and afferent arterioles following UUO. The HIF-2α staining pattern shifting from a cytoplasmic subcellular location before UUO to a nuclear location HIF-2α on day 3 post UUO is very suggestive of HIF-2α changing from its inactive form to an active form. Although this phenomenon preceded the increase in CoRL number in the intersitium, activated HIF2a persisted in a subset of CoRL at all time points studied. There is precedence for HIF in CoRL. Kurtz et al. mimicked chronic hypoxia in CoRL by the deletion of Hippel-Lindau protein, and showed increased HIF-2α expression along the afferent arterioles, glomerular vascular poles, and intraglomerular cells [15]. Also perhaps relevant to the current studies is that the chronic activation of HIF-2α transforms a subset of cells in the JG compartment into fibroblasts-like cells [47]. Taken together, we propose that a likely mechanism that underlies the transdifferentiation of CoRL in the JG following UUO is the activation of HIF-2α. Further studies are needed to prove if HIF-2α favors a pericyte-like and/or myofibroblast-like transdifferentiation of CoRL in UUO.

This study has some limitations. First, the study is largely descriptive as we focus on association between HIF-2α and CoRL activation. However there is sufficient literature to support our proposed claim that HIF-2α may be a causative factor leading to CoRL migration and proliferation. JG area has been shown to display marked plasticity in disease settings [13–16]. Also, developmental studies demonstrate that arterioles are the source of pericyte recruitment [48]. Second, UUO model does not provide functional data [49, 50]. One may argue that only a small number of CoRL is involved in vascular remodelling in UUO, however in an inducible fate-mapping approach only a fraction of cells is labelled (as we observe glomeruli without labelled CoRL). Importantly, the strength of this approach is that it allows to faithfully track the subpopulation of CoRL that were permanently labeled during tamoxifen induction. Finally, it adds to the pool of known pro-fibrotic progenitors arteriolar-derived myofibroblasts. Third limitation of the study is a gender bias since we have only used female mice. Most of the studies use male mice and rats and the pro-injury effect of androgens in renal injury in males is well documented [51, 52]. Nevertheless, in our UUO model females demonstrated the classical features of renal injury.

## Conclusions

In summary, this study demonstrated that CoRL are resident kidney progenitors that can respond to vascular injury by proliferation and migration to possibly participate in vessel remodeling. However, with time, CoRL ultimately undergo transition into myofibroblast-like cells, which might favor fibrosis rather than repair. The role of HIF-2a in CoRL needs further exploration.

## Additional files

Additional file 1: Figure S1. UUO induces kidney fibrosis and reduces vascular density in Ren1cCre mice. (**A**) Following ureteral obstruction of the right kidney, the ureter is filled with urine. Non-obstructed kidney (contralateral) undergoes hypertrophy. (**B**) Reduced weight ratio of obstructed/non-obstructed kidney on day 7 and 14 after UUO. (**C**) Endothelial cells, identified by CD31 staining (green), decrease in the cortex and medulla following UUO on day 7 and 14 compared to sham kidneys. Examples of capillary rarefaction are marked with an asterisk (*). (**D**) Picrosirius Red staining was examined by polarized light microscopy. Collagen fibers were barely detectable in sham kidneys. Interstitial Picrosirius Red staining was increased on days 3, 7 and 14 following UUO. (**E**) Fibrosis quantification based on Picrosirius Red staining score shows a gradual increase of fibrosis in UUO. Data are represented as mean ± SEM. (PNG 652 kb) Additional file 2: Figure S2. Renin staining is limited to labeled CoRL in JG and afferent arterioles. Red fluorescent protein staining identified CoRL (red), renin staining indicates renin-producing cells (green). DAPI (blue) staining labels nuclei. At 7 days post UUO, (**A**) in the cortex, renin staining was detected in classical JG location (arrow) (**B**) in the medulla there was no renin staining found, interstitial CoRL were negative for renin. (PNG 431 kb) Additional file 3: Figure S3. HIF-2α is not activated in interstitial CoRL. Red fluorescent protein (red) staining identified CoRL, HIF-2α (green) staining was used to detect hypoxia-activated cells. Nuclei are labeled with DAPI (blue). (**A**) Sporadic HIF-2α-expressing cells were found in sham kidney. Following UUO, there was an increase of HIF-2α-activated cells (arrowheads) at (**B**) d3, (**C**) d7, (**D**) and d14. However, none of interstitial CoRL expressed HIF-2α (arrows). (PNG 1063 kb)

## Abbreviations

CoRL: cells of renin lineage
HIF-2α: hypoxia inducible factor-2α
JG: juxtaglomerular
PDGFß-R: platelet derived growth factor Beta receptor
RFP: red fluorescent protein
TR: tomato red
UUO: unilateral ureteral obstruction
