# Supplementary figures and images for: Cells of renin lineage express hypoxia inducible factor 2α following experimental ureteral obstruction
Source: BMC Nephrol. 2016 Jan 8;17:5. doi: 10.1186/s12882-015-0216-0 (PMC4706659; doi:10.1186/s12882-015-0216-0)

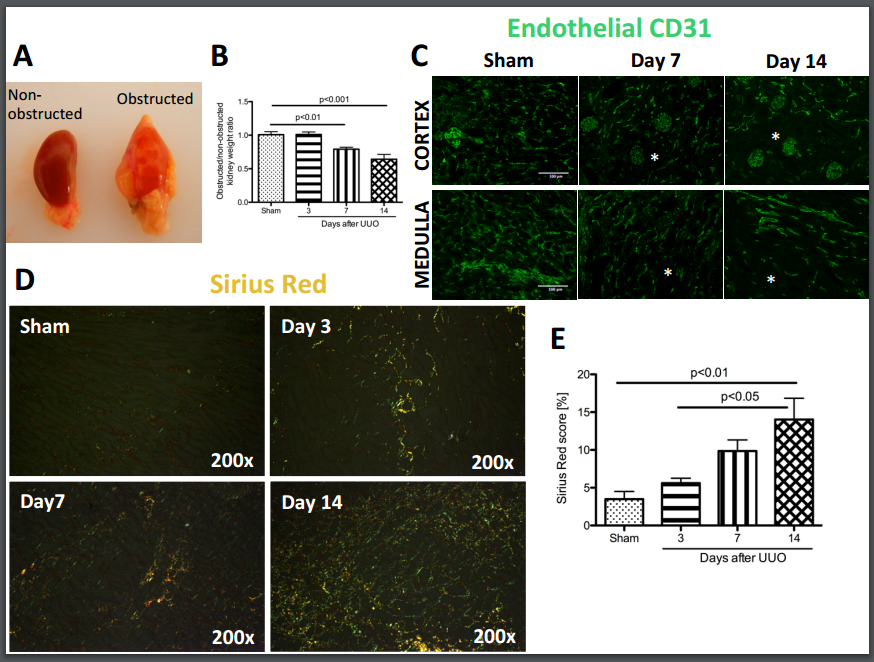

Supplement: Additional file 1: Figure S1. — UUO induces kidney fibrosis and reduces vascular density in Ren1cCre mice. (A) Following ureteral obstruction of the right kidney, the ureter is filled with urine. Non-obstructed kidney (contralateral) undergoes hypertrophy. (B) Reduced weight ratio of obstructed/non-obstructed kidney on day 7 and 14 after UUO. (C) Endothelial cells, identified by CD31 staining (green), decrease in the cortex and medulla following UUO on day 7 and 14 compared to sham kidneys. Examples of capillary rarefaction are marked with an asterisk (*). (D) Picrosirius Red staining was examined by polarized light microscopy. Collagen fibers were barely detectable in sham kidneys. Interstitial Picrosirius Red staining was increased on days 3, 7 and 14 following UUO. (E) Fibrosis quantification based on Picrosirius Red staining score shows a gradual increase of fibrosis in UUO. Data are represented as mean ± SEM. (PNG 652 kb) [file 12882_2015_216_MOESM1_ESM.png]

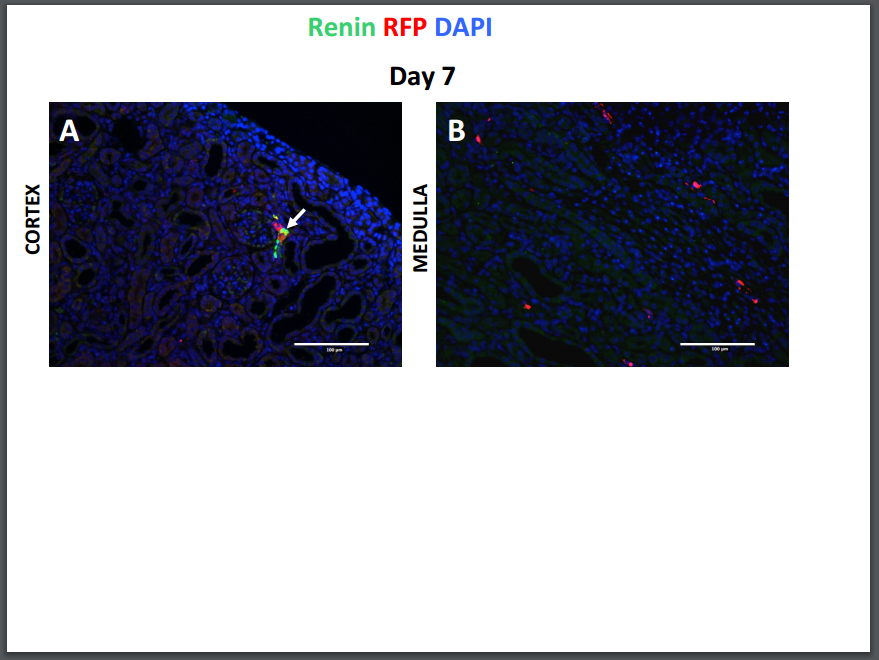

Supplement: Additional file 2: Figure S2. — Renin staining is limited to labeled CoRL in JG and afferent arterioles. Red fluorescent protein staining identified CoRL (red), renin staining indicates renin-producing cells (green). DAPI (blue) staining labels nuclei. At 7 days post UUO, (A) in the cortex, renin staining was detected in classical JG location (arrow) (B) in the medulla there was no renin staining found, interstitial CoRL were negative for renin. (PNG 431 kb) [file 12882_2015_216_MOESM2_ESM.png]

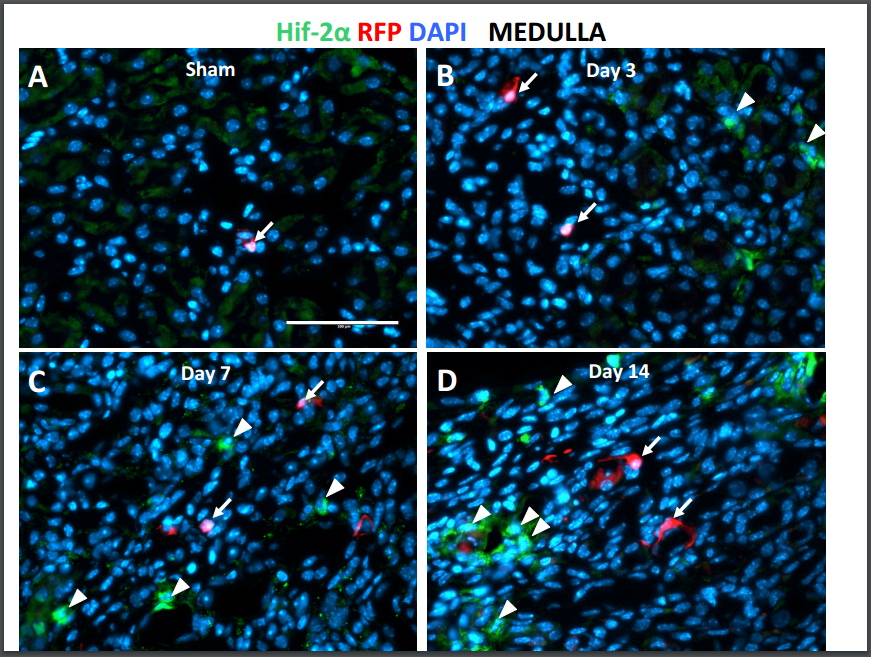

Supplement: Additional file 3: Figure S3. — HIF-2α is not activated in interstitial CoRL. Red fluorescent protein (red) staining identified CoRL, HIF-2α (green) staining was used to detect hypoxia-activated cells. Nuclei are labeled with DAPI (blue). (A) Sporadic HIF-2α-expressing cells were found in sham kidney. Following UUO, there was an increase of HIF-2α-activated cells (arrowheads) at (B) d3, (C) d7, (D) and d14. However, none of interstitial CoRL expressed HIF-2α (arrows). (PNG 1063 kb) [file 12882_2015_216_MOESM3_ESM.png]
